# Topological Design of Two-Dimensional Phononic Crystals Based on Genetic Algorithm

**DOI:** 10.3390/ma16165606

**Published:** 2023-08-13

**Authors:** Xiaodong Wen, Lei Kang, Xiaowei Sun, Ting Song, Liangwen Qi, Yue Cao

**Affiliations:** School of Mathematics and Physics, Lanzhou Jiaotong University, Lanzhou 730070, China

**Keywords:** phononic crystals, adaptive genetic algorithm, finite-element method, maximum relative bandwidth, low-frequency bandgap

## Abstract

Phononic crystals are a kind of artificial acoustic metamaterial whose mass density and elastic modulus are periodically arranged. The precise and efficient design of phononic crystals with specific bandgap characteristics has attracted increasing attention in past decades. In this paper, an improved adaptive genetic algorithm is proposed for the reverse customization of two-dimensional phononic crystals designed to maximize the relative bandwidth at low frequencies. The energy band dispersion relation and transmission loss of the optimal structure are calculated by the finite-element method, and the effective wave-attenuation effect in the bandgap range is verified. This provides a solution for the custom-made design of acoustic metamaterials with excellent low-frequency bandgap sound insulation or other engineering applications.

## 1. Introduction

Mechanical vibration is a widespread physical phenomenon that is commonly encountered in the field of mechanical engineering. It exists in various degrees in military equipment such as military aircraft, submarines, warships, missiles, as well as in everyday transportation vehicles like trains and cars [1]. In practical industrial applications, mechanical vibration can reduce equipment productivity and lifespan in mild cases, and pose a threat to industrial production and safety in severe cases [2]. Vibration is usually accompanied by noise generation. Prolonged noise exposure not only seriously affects people’s quality of life, but also damages nerve cells and auditory tissue [3].

Numerous experimental studies have shown that the noise generated by mechanical vibration is mainly distributed in the low-frequency range from 20 to 500 Hz [4,5,6]. The distinguishing features of low-frequency noise are its strong penetration capability, slow decay, and susceptibility to masking by mid-to-high-frequency noise [7]. For example, the wavelength of low-frequency noise in the air at 20 Hz is about 17 m. In traditional vibration and noise-reduction devices, such as sound barriers, the required thickness of the sound barrier is far greater than the size of general building wall structures and does not have practicality. Therefore, it is difficult to effectively suppress low-frequency vibration and noise using traditional vibration and noise-reduction devices [8,9].

In recent years, with the extensive research on artificial acoustic metamaterials, new approaches and methods have emerged to address the challenges of low-frequency vibration and noise control, which benefit from the unique properties of artificial acoustic metamaterials as opposed to natural materials [10,11,12]. Phononic crystals, as a significant branch of artificial composite periodic structures with distinct characteristics have demonstrated excellent control capabilities over mid-to-low-frequency elastic waves. By utilizing the principle of “small size controlling large wavelengths”, phononic crystals offer a promising avenue for controlling low-frequency vibrations and noise [13,14,15,16,17].

However, despite the favorable sound-insulation performance exhibited by phononic crystals at different elastic wave frequencies, the currently designed phononic crystal structures tend to have relatively large dimensions, which are typically on the order of tens of millimeters. In the low-frequency range, particularly below 500 Hz, the optimization of sound-insulation performance for practical applications remains a challenge that has yet to be effectively addressed [18,19,20,21,22,23,24,25,26,27,28,29,30]. For engineering applications, obtaining the widest possible bandgap within the desired frequency range is a key issue in the design of phononic crystal structures. The bandgap structure of phononic crystals can also be designed and customized by changing the filling rate and using new materials with different elastic constants. However, using topology optimization techniques to obtain the optimal periodic lattice topology shape that produces the maximum bandgap width in phononic crystals has undoubtedly become an important approach [31,32]. Compared to traditional trial-and-error methods, a systematic scientific approach involves formulating the problem mathematically and then solving it through topology optimization [33]. For typical two-dimensional phononic crystals, topology optimization of the bandgap using genetic algorithms allows for the reconfiguration of material components within the existing phononic crystal structure. This can lead to the realization of model structures that were previously unimaginable with conventional design approaches, while also avoiding the tediousness and uncertainty associated with the manual design of phononic crystal structures. In addition, other optimization algorithms similar to genetic algorithms, such as simulated annealing algorithms, particle swarm algorithms, and artificial immunity algorithms can be used to solve discrete optimization problems. Molecular dynamics, which is an effective tool for studying the structure of microscopic materials, can also be used to search and optimize phononic crystals [34,35,36,37]. This enables the development and application of functional devices related to phononic crystals. In recent years, Zhao et al. proposed a pattern-recognition algorithm that combines polar microstructure surface features [38]. Deng et al. proposed a power-allocation-ratio calculation method for adaptive power allocation based on an artificial neural-network model [39]. As these machine-learning algorithms continue to evolve and be applied in multiple domains, this also provides an effective method for the inverse customized design of phononic crystals.

Topology optimization originated in structural engineering, looking for the distribution of materials in each region to achieve the best or desired structural properties. In earlier years, researchers have designed complex materials, such as cluster assembly materials, using DFT and other ab initio theoretical methods applied to actual chemical composition [40,41]. Zhang et al., proposed and demonstrated a scheme to realize a Luneburg lens on a chip through the integration of gradient metamaterial structure and silicon waveguides [42]. Since the pioneering work of Bendsøe and Kikuchi in 1988 [43], various gradient-based topology optimization methods have been proposed, such as solid isotropic material with penalization (SIMP) [44,45], level-set methods, and bi-directional evolutionary structural optimization [46,47,48,49,50]. Non-gradient-based topology optimization methods such as genetic algorithms have also been widely applied in structural topology optimization [51]. The topology optimization of phononic bandgap structures was first realized by Sigmund and Jensen in 2003 using finite-element methods combined with the method of moving asymptotes to maximize the bandgap [52]. This pioneering work lacked analysis of the coupling between in-plane waves and out-of-plane waves. Subsequently, Gazonas et al. combined genetic algorithms with finite-element methods and proposed a method for optimizing out-of-plane waves in two-dimensional phononic crystals [53]. Hussein et al. performed a series of optimizations on one-dimensional and two-dimensional phononic crystals using genetic algorithms [54,55]. Bilal et al. developed a gradient-based topology optimization approach to design two-dimensional and three-dimensional phononic filters, including surface waveguides [56]. Dong et al., conducted a more detailed work on topology optimization of two-dimensional phononic crystals, utilizing a two-level genetic algorithm and finite-element method to optimize out-of-plane waves and in-plane waves with or without volume constraints [57]. Liu et al. used a two-level genetic algorithm combined with the plane-wave-expansion method to optimize the bandgap widths of in-plane coupled modes, out-of-plane acoustic modes, and hybrid modes in phononic crystals [58].

## 2. Simulation Models and Calculation Methods

**Figure 1:**
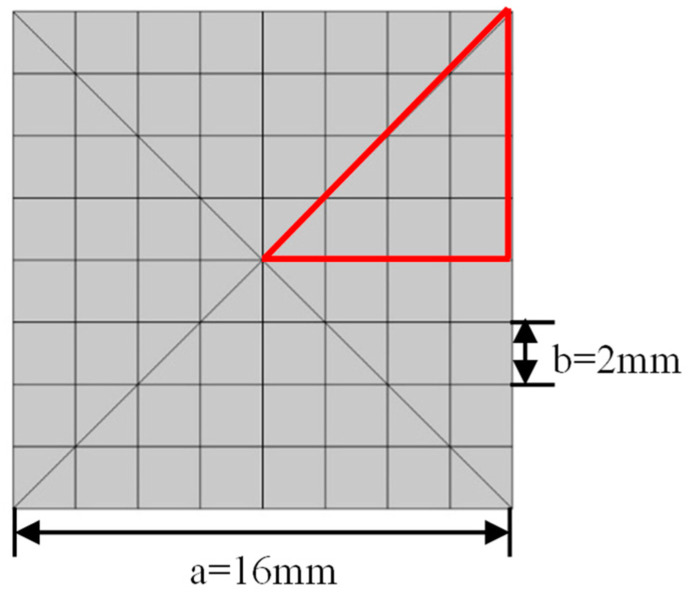
Schematic diagram of the single-cell structure and geometric parameters of phononic crystal plates.

The ideal two-dimensional phononic crystal structure is formed by the sequential arrangement of an infinite number of periodically repeated unit cells in the spatial plane, which are periodic in both the x and y directions. Studies have shown that the bandgap of a phononic crystal is mainly influenced by structural dimensions, oscillator coupling strength, and the softness of the elastic cladding layer. Therefore, the choice of different materials for the matrix, oscillators and elastic cladding layer plays a crucial role in determining the bandgap. To seek a maximum relative bandwidth in a smaller phononic crystal geometric size and accelerate the convergence of calculations, this study proposes a square lattice structure for the two-dimensional phononic crystal plate unit-cell model. As shown in Figure 1, the lattice constant is set to 16 mm and is divided into an 8 × 8 grid with a total of 64 small squares, each of which is 2 mm in size. Each hole in the lattice structure can be filled with two different densities of materials. The material parameters used in this study are given in Table 1 below.

In general, a phononic crystal composed of various isotropic elastic materials can be regarded as a heterogeneous elastic material. Here, we only consider the propagation of elastic waves in a two-dimensional periodic structure. According to the theory of elasticity, the governing equation for the propagation of elastic waves in a two-dimensional non-uniform elastic continuum can be expressed as follows:

(1) $$\nabla \left\{\left[\lambda \left(r\right)+2\mu \left(r\right)\right]\nabla \cdot u\right\}-\nabla \left[\mu \left(r\right)\nabla u\right]+\rho \omega^{2}u=0$$

where ***r*** = (*x*, *y*, *z*) is the position vector, *λ* and *μ* are the Lamé constants, ***u*** = (*u_x_*, *u_y_*) is the transverse plane displacement vector, *ρ* is the mass density, and *ω* is the angular frequency.

For the in-plane mixed mode, the equation can be rewritten as

(2) $$\nabla \cdot \left[\mu \left(r\right)\nabla u_{e}\right]+\nabla \cdot \left[\mu \left(r\right)\frac{\partial }{\partial x_{e}}u\right]+\frac{\partial }{\partial x_{e}}\left[\lambda \left(r\right)\nabla \cdot u\right]+\rho \left(r\right)\omega^{2}u_{e}=0$$

In an ideal phononic crystal with discrete translational symmetry, satisfying the Bloch theorem for periodic systems, the displacement at each point can be represented as

(3) $$u\left(r\right)=e^{i\left(k\cdot r\right)}u_{k}\left(r\right)$$

here ***u_k_***(***r***) is a periodic vector function with the same periodicity as the lattice, $k=\left(k_{x+u_{x}},k_{y+u_{y}}\right)$ is the wavevector of the Bloch wave selected only within the first Brillouin zone.

In the unit cell, the generalized eigenvalue equation can be represented as

(4) $$\left(K-\omega^{2}M\right)U=0$$

where ***U*** the characteristic tensor, ***K*** the stiffness tensor, and ***M*** the mass tensor.

The displacement field satisfies the Bloch periodicity condition on two opposite boundaries of the unit cell and can be expressed as

(5) $$u\left(r+a\right)=e^{i\left(k\cdot a\right)}u_{k}\left(r\right)$$

here ***r*** is the position vector at the boundary nodes, and ***a*** is the lattice basis vector.

By introducing the concept of the Brillouin zone, the first Brillouin zone is often referred to as the irreducible Brillouin zone because the volume of the nth Brillouin zone is the same as that of the first Brillouin zone. According to the band theory, to calculate bandgaps, periodic boundary conditions are applied in the *x* and *y* directions of the boundaries of the two-dimensional phononic crystal unit cell within the integrable Brillouin zone. The material filling of the unit-cell design domain is performed using a developed solid mechanics program module, and a subroutine module is called to scan the Bloch wave vector within the range of 0 to 3 over the high-symmetry boundaries of the irreducible Brillouin zone. For the eigenvalue equation in Equation (4), it is only necessary to solve for the Bloch wave vectors on the boundaries of the irreducible Brillouin zone. As shown by the red triangle in Figure 1, the corresponding eigenfrequency *ω* can be calculated to obtain the corresponding band dispersion relation *ω*(***k***) along the path Γ→X→M→Γ.

## 3. Topological Optimization Design of Binary Microstructural Phononic Crystals

### 3.1. Algorithmic Description of Binary Materials

In previous studies, genetic algorithms have demonstrated high efficiency in the optimization design of two-dimensional phononic crystals. This paper discusses a two-dimensional binary phononic crystal thin plate. The matrix material is chosen as a low-density soft material, with density, Young’s modulus, and Poisson’s ratio represented by *ρ_a_*, *E_a_* and *μ_a_*, respectively. The scatterer is selected as a high-density material, with corresponding density, Young’s modulus, and Poisson’s ratio denoted by *ρ_b_*, *E_b_* and *μ_b_*, respectively. The selected material parameters are shown in Table 1. For each element in the 8×8 unit cell structure, the material parameters can be defined as follows:

(6) $$\rho \left(x_{i}\right)=\left(1-x_{i}\right)\rho_{a}+x_{i}\rho_{b}$$

(7) $$E\left(x_{i}\right)=\left(1-x_{i}\right)E_{a}+x_{i}E_{b}$$

(8) $$\mu \left(x_{i}\right)=\left(1-x_{i}\right)\mu_{a}+x_{i}\mu_{b}$$

here $i\in \left[1,2,3,\cdots ,64\right]$, and *x_i_* take values of either 0 or 1.

In the genetic algorithm, each chromosome represents a specific solution to the problem and each chromosome consists of multiple genes. Each gene represents a decision variable of the solution. In the specific optimization design process for the 2D binary square lattice phononic crystal plate, the chromosome-encoding problem involves dividing the unit cell into *N* × *N* pixels to construct an *N* × *N* logical matrix. Each element of the matrix takes a value of 0 or 1, where binary 0 and 1, respectively, represent low-density and high-density materials. Thus, there is a one-to-one mapping between the unit cell’s pixels and the binary digits 0 or 1 on the gene positions of the chromosome. Consequently, each square lattice phononic crystal plate cell in the studied problem can be viewed as a sequential arrangement of binary digits 0 or 1 in the *xy*-plane. To reduce computational costs, it can be assumed that the square lattice is symmetric along the *x* and y axes and has four-fold rotational symmetry along the *z*-axis. On this basis, the optimal design problem for a single-cell structure is simply a matter of determining one-eighth of the entire square structure, as shown in the red triangular region in Figure 1. This implies that the dimensionality of the phenotype of an individual can be reduced from *N* × *N* to $\frac{\left(N/2\times N/2\right)}{2}+N/4$.

### 3.2. Improved Genetic Algorithm

Different from conventional optimization methods, genetic algorithms utilize the cost function itself to search for the optimal solution from multiple parallel points. This means that genetic algorithms are more suitable for achieving global optimality for problems that may have many local optima [59]. Depending on the application, the search space of genetic algorithms can be divided into continuous space and discrete space, which are called continuous and discrete genetic algorithms, respectively. In discrete genetic algorithms, a commonly used method for individual encoding is finite-length binary coding, which has the advantage of facilitating the establishment of mathematical models for genetic algorithms but is not convenient for human expression. As described in the previous section on the algorithmic description of binary two-component gametes, once the correspondence between the cell-filling materials, pixels, and binary codes is established, changing the binary encoding of each gene in the chromosome can construct different phenotypic cell structures. This indicates that genetic algorithms based on binary coding are more suitable for the topological optimization problem of discrete two-dimensional square-lattice phononic crystals.

**Figure 2:**
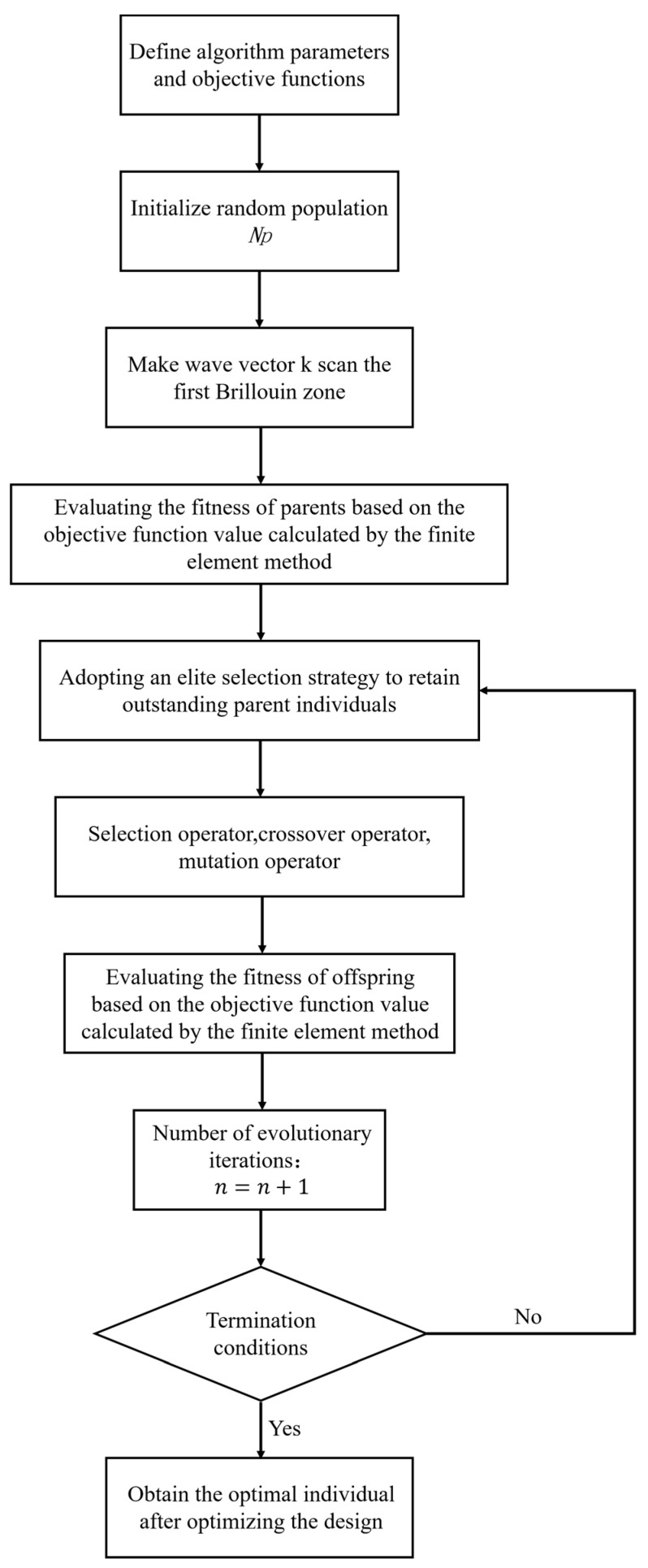
The flow chart of filling-material distribution algorithm optimization design.

In phononic crystals, the spatial distribution of constituent materials plays a crucial role in the generation of bandgaps. Therefore, in order to achieve the maximum relative bandwidth in various frequency ranges, it is necessary to design the filling distribution of materials. The finite-element method is employed to calculate the band structure. The improved adaptive genetic algorithm is used to determine the optimal filling distribution of materials [60]. The algorithmic optimization design process for the filling material distribution is illustrated in Figure 2.

The optimization objective here is to obtain an optimized unit cell model with the maximum relative bandwidth between two adjacent frequency bands. Therefore, this optimization problem can be described mathematically as an objective function:

(9) $$\begin{array}{c} \mathrm{Maximize}:f=\underset{n=1,2\ldots }{\max}\left(2\frac{\underset{k}{\min}:\omega_{n+1}\left(k,X\right)-\underset{k}{\max}:\omega_{n}\left(k,X\right)}{\underset{k}{\min}:\omega_{n+1}\left(k,X\right)+\underset{k}{\max}:\omega_{n}\left(k,X\right)}\right)\\ \mathrm{Subject}\mathrm{to}:x_{i}=0\mathrm{or}1 \end{array}$$

where *f* represents the maximum relative bandwidth, which is dimensionless and simplified from the center frequency between a given pair of adjacent frequency bands. ***X*** represents the chromosome of the topology optimization problem under study and ***k*** is the wave vector. The order of the bandgap in the objective function is extracted based on *n* and *ω_n_* denotes the frequency of the nth band. The iterative evolution process for solving the optimization problem in Equation (9) using the improved genetic algorithm is as follows:

(1) The generation of the initial population.

The initial population, represented as the chromosome matrix Chrom, consisting of *N_p_* random individuals, is created using a binary encoding. For an 8 × 8 square lattice of a two-dimensional phononic crystal unit cell, the dimensionality of the phenotype (individual representation) is 10, which corresponds to the number of decision design variables for each individual chromosome. In most cases, a random structure does not exhibit a bandgap, but it helps converge to an approximate global optimum. The chromosome matrix Chrom is organized such that each row corresponds to a chromosome of an individual, and each chromosome stores the information of the decision variables representing the material filling of the unit cell. The matrix representation is as follows:

(10) $$\mathrm{Chrom}=\left[\begin{array}{cccccccccc} 1 & 1 & 0 & 1 & 1 & 0 & 0 & 1 & 1 & 1\\ 0 & 1 & 1 & 0 & 1 & 0 & 0 & 1 & 1 & 1\\ 1 & 0 & 1 & 1 & 1 & 1 & 1 & 0 & 1 & 0\\ \ldots & & & & & & & & & \end{array}\right]$$

(2) The fitness functions.

The fitness evaluation function reflects the individual’s adaptability to the environment. The lower the value f of the objective functions, the stronger the adaptability. To conform to common practice, the problem of minimizing *f* is transformed into maximizing—*f*. Therefore, the fitness evaluation function is given by—*f*, where a higher value indicates stronger adaptability and should be preserved. Two individuals are randomly selected and their fitness values are compared. The individual with higher fitness is selected as the parent.

(3) The determination of genetic operators.

For the selection step, the roulette-wheel selection algorithm, primarily used in optimization algorithms, is adopted. In this method, the selection probability of individuals is directly proportional to its fitness value. Additionally, to avoid getting trapped in local optimization during the algorithm’s iteration process, an elitism strategy is introduced. This means that the individual with the highest fitness value is preserved in each selection step. This optimization helps improve the quality of the population and enhances the stability and convergence of the algorithm.

For the crossover step, a single-point crossover is used. The crossover operator probability *P_c_* is set to 0.70. Crossover is applied to everyone independently. If a randomly generated probability is less than 0.70, the individual can randomly exchange genetic information with another individual to generate new offspring.

For the mutation step, a single-point mutation is used. The mutation operator probability *P_m_* is set to 0.02. A mutation is applied to each gene of the chromosome. If a randomly generated probability is less than 0.02, the gene undergoes mutation by swapping the binary values of 0 and 1.

Through the application of these genetic operators, the diversity and partial superiority of the resulting offspring population are ensured.

(4) The termination conditions.

Repeat steps (2) to (3) iteratively, with a maximum of 100 generations, until the convergence condition is met. If the improvement in the best value after 85 generations does not exceed 1 × 10^−6^, the algorithm will be terminated in advance. This allows for a balance between the execution time of the algorithm and its accuracy.

## 4. Results and Discussions

### 4.1. Transmission Loss

**Figure 3:**
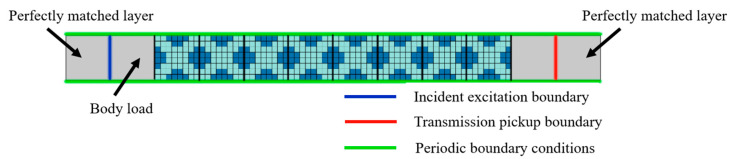
Finite-element simulation model for calculating transmission spectrum.

We constructed a finite phononic crystal long plate structure with eight-unit cells along the wave-propagation direction, as shown in Figure 3. In this case, we assume that the wave propagates along the *x* direction. Therefore, periodic boundary conditions are applied at the top and bottom edges in the *y* direction. To avoid interference from wave reflections, the left and right boundaries of the long plate structure are set as perfect matching layers. A vertically incident elastic wave excitation is applied at the left boundary of the structure and the displacement of the wave propagating to the right is measured at the right boundary. The transmission loss (T) of the structure is obtained by calculating the difference between the time average power at the incident boundary and transmiassion pickup boundary, as shown in Figure 3, with equation expressed as [61]:

(11) $$T=10\log_{10}\left(\frac{W_{out}}{W_{in}}\right)$$

where *W_in_* and *W_out_* are the time average incident power and transmitted power.

We performed simulation calculations using the finite-element method on an 8 × 8 long plate structure composed of a two-dimensional square-lattice phononic crystal unit cell. The material properties, boundary conditions, and applied loads were defined using the solid mechanics module. An initial planar force of 10 N was applied to the finite plate structure. In the subsequent sections, the acoustic isolation performance of the optimized unit-cell structures was analyzed using the finite element transmission loss simulation model shown in Figure 3. The only difference is that we changed the spatial distribution structure of the filling materials in the model, i.e., we replaced the eight-unit cell structures in the model.

For most phononic crystal topology optimization problems, the emphasis lies in seeking the maximum relative bandwidth within the range of elastic wave frequencies. However, two issues often arise: Firstly, the optimized structure may not be the global optimum due to the incomplete search space. Secondly, the optimized structure may lack practical engineering or mechanical application value. Therefore, starting from the design of the initial structure and the direction of algorithm optimization, we analyze the optimization problem of small-sized square-lattice phononic crystal thin-plate structures as follows:

(1) Materials with large impedance differences are more likely to exhibit bandgaps.
(2) According to the band-gap mechanism of phononic crystals, it is known that the center frequency of the lowest bandgap generated by Bragg-type phononic crystals is approximately *c*/2*a*, where c is the elastic wave velocity and the lattice size. Therefore, phononic crystals based on Bragg scattering mechanisms are more likely to generate medium-to-high frequency bandgaps, while localized resonance mechanisms, with their ability to control large wavelengths through small dimensions, are more likely to open bandgaps at lower frequencies.
(3) For locally resonant phononic crystals, the frequency range can be controlled by adjusting the effective spring constant of the unit-cell structure and the block mass of the scatterers, as indicated by the frequency formula of the spring-mass oscillator model.

(12) $$f=2\pi \sqrt{\frac{k}{m}}$$

According to the above discussion, the article presents two examples in different frequency ranges of elastic waves, analyzing how to effectively optimize the phononic crystal thin-plate structure within the desired frequency range.

### 4.2. Case 1: Maximizing Relative Bandwidth in the Specified Mid–Low Frequency Range

In this case, the objective is to construct a phononic crystal thin-plate structure that is both engineering practical and exhibits a wide relative bandgap in the mid–low frequency range. To lower the position of the starting frequency, it is necessary to enhance the local resonance effect and reduce the influence of the Bragg scattering mechanism. This can be achieved by increasing the mass of the scattering bodies in the central region of the structure. Therefore, the optimization process of the above algorithm needs to add the following constraints to the objective function of Equation (9):

(13) $$\begin{array}{ll} \mathrm{Maximize}: & f\\ & \omega_{n}(k,X)>200\;\mathrm{Hz}\\ & \omega_{n+1}(k,X)<1500\;\mathrm{Hz}\\ & \frac{1}{3}A<C_{0}<\frac{5}{6}A \end{array}$$

In the above equation, *A* is the designated internal central area of 6 × 6 square grid areas and *C*_0_ represent the filling rate of scatterers in the central region.

**Figure 4:**
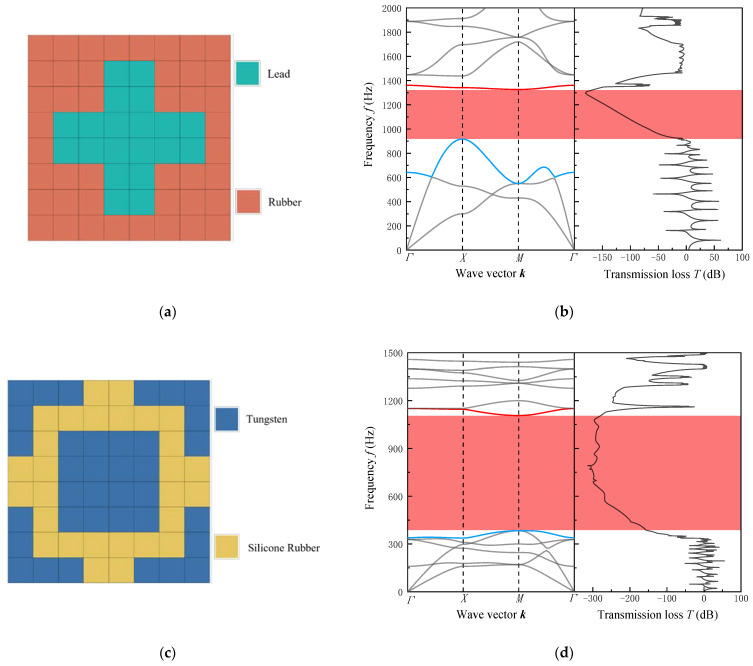
(**a**,**c**) Single-cell structure; (**b**,**d**) energy-band dispersion relation and transmission spectrum for Case 1.

As shown in Figure 4, structure A can be regarded as consisting of a “cross-shaped” lead scatterer surrounded by the base material rubber, with a bandgap ranging from 930 Hz to 1300 Hz. Structure B is formed by connecting the silicon rubber enveloping the “square” tungsten block at the center with tungsten material at the four corners, and it exhibits a bandgap ranging from 388 Hz to 1108 Hz. By comparing the constituent materials and the size range of the bandgaps produced by these two structures, it can be observed that materials with greater density differences have lower starting frequencies and wider bandwidths. Meanwhile, the two optimal structures obtained for the mid–low frequency range have larger distributions of scatterer solid block mass in the central region of the square-lattice phononic crystal thin plate. This further validates our assumptions and analysis of the optimization problem and the reliability of the algorithmic optimization approach.

In general, the resonance peaks of highway traffic noise are concentrated around 500 Hz, while the resonance peaks between car tires and road surfaces are concentrated around 1000 Hz. From Figure 4b,d of Structure A and Structure B, it can be observed that both structures exhibit effective sound-pressure attenuation within the bandgap range. Therefore, the optimized structures mentioned above can provide a new design concept for noise reduction applications in highway sound barriers.

### 4.3. Case 2: Maximizing Relative Bandwidth in the Specified Ultra-Low Frequency Range

Based on the analysis of the previous example, the effectiveness and advantages of combining the finite-element method with the genetic algorithm for phononic-crystal-structure optimization have been further validated. Previous studies have focused on optimizing phononic crystal thin-plate structures, but due to the inherent limitations of simple thin-plate structures, it is difficult to achieve wide bandgaps in the ultra-low frequency range.

To open wide bandgaps at low frequencies, a constrained algorithm optimization approach can be applied to the thin-plate structure, considering both the overall structure and sub-regions. Firstly, to avoid weakening the local resonance effect of the thin plate, it is necessary to control the mass of the central scatterer, which can also prevent the algorithm from overly optimizing toward the Bragg scattering mechanism. Secondly, high-density materials are placed at the four corners of the square lattice, and these corner materials should not be connected to the central scatterer, effectively achieving the equivalent effect of serially connected springs and reducing the stiffness coefficient of the soft material. Thirdly, the filling ratio of scatterers and soft materials in the overall structure needs to be controlled to enhance the local resonance effect and better generate low-frequency bandgaps.

Therefore, to maximize the relative bandwidth in the low-frequency range, the objective function remains the same, but additional constraints are added. The specific form of the algorithm is as follows.

(14) $$\begin{array}{ll} \mathrm{Maximize}: & f\\ & \omega_{n}(k,X)>5\;\mathrm{Hz}\\ & \omega_{n+1}(k,X)<150\;\mathrm{Hz}\\ & 0.0625\leq C_{1}\leq 0.4375\\ & 0.0625\leq C_{2}\leq 0.2500\\ & 0.1250\leq C_{3}\leq 0.6875 \end{array}$$

In the above equation, *C*_1_, *C*_2_, and *C*_3_ represent the filling ratios of scatterers inside the central region, at the four corners, and in the overall structure, respectively, for the square lattice thin-plate unit cell.

**Figure 5:**
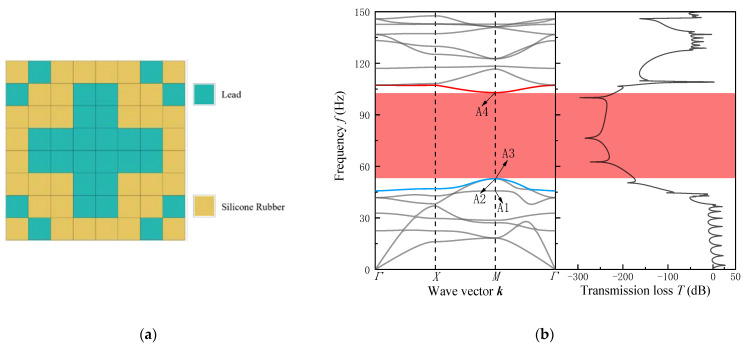

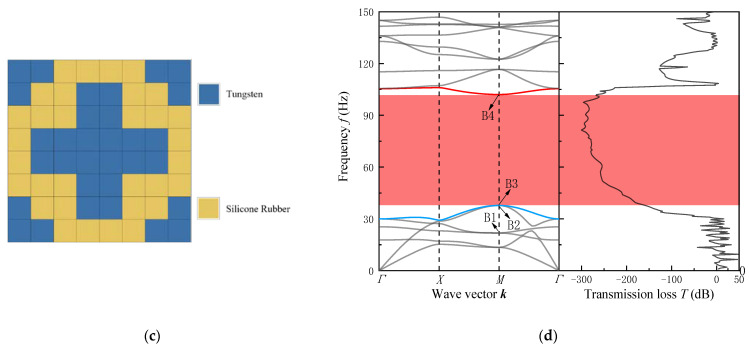
(**a**,**c**) Single-cell structure; (**b**,**d**) energy-band dispersion relation and transmission spectrum for Case 2.

The optimized model’s unit-cell structure, band-dispersion relation, and transmission spectrum are shown in Figure 5. From the obtained structures, it can be observed that the designed structures can be seen as composed of solid blocks distributed at the center and four corners, connected by a soft material. At the center of the structure, the topology can be visualized as a “cross” structure.

**Figure 6:**
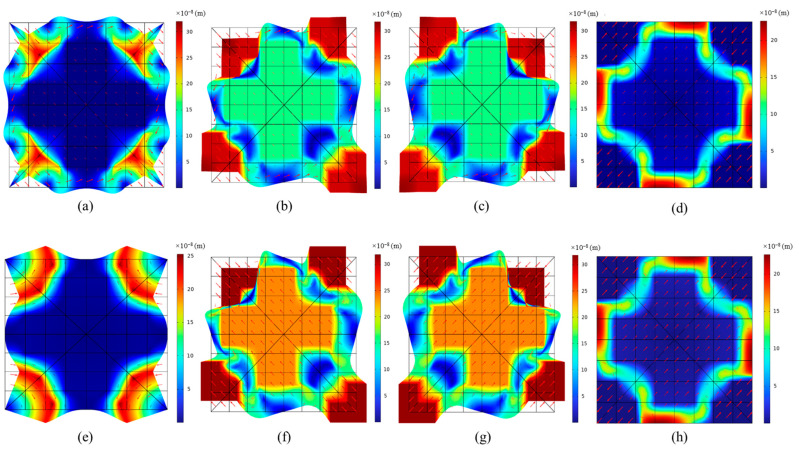
Vibration mode diagrams of model A and model B: (**a**) Mode at point A1 (f = 45.710 Hz). (**b**) Mode at point A2 (f = 52.816 Hz). (**c**) Mode at point A3 (f = 52.837 Hz). (**d**) Mode at point A4 (f = 102.940 Hz). (**e**) Mode at point B1 (f = 21.951 Hz). (**f**) Mode at point B2 (f = 37.755 Hz). (**g**) Mode at point B3 (f = 37.773 Hz). (**h**) Mode at point B4 (f = 102.060 Hz).

The frequency ranges of structures A and B, respectively, are 52.8 Hz to 102.9 Hz and 37.7 Hz to 102.1 Hz. The bandgap range of the two structures achieves a good match with the constraints of the genetic algorithm optimization function designed in the paper. The comparison of structures A and B shows that the soft material of both structures is silicone rubber, while the scatterer material of structure B is tungsten. The first bandgap onset frequency of structure B is about 15 Hz lower than that of structure A. According to the local resonance mechanism, this is due to the large impedance difference between the constituent materials of the structure. Additionally, from the transmission spectra in Figure 5b,d of the two structures, both structure A and structure B achieve a sound-pressure attenuation of less than 200 dB over the bandgap range. It can be observed that there is significant attenuation in the corresponding low-frequency bandgaps. Compared to the reported three-component tough bandgap phononic crystal thin plates by Cheng et al. [60], it is found that the two obtained optimal structures both exhibit continuous and complete low-frequency wide bandgaps within the first bandgap range. By analyzing the vibration modes near the starting and ending frequencies of the first bandgap for Structure A and Structure B in Figure 6, it can be clearly observed that torsional resonance occurs at the starting position of the low-frequency bandgap, and there is a reverse resonance between the scatterers and the matrix at the ending position of the bandgap. Some vibration modes exhibit high symmetry, indicating that the low-frequency bandgaps opened by the two structures conform to the locally resonant mechanism. This further validates the correctness of the theory of the optimization hypothesis analysis based on the local resonance mechanism in the paper.

## 5. Conclusions

This study focuses on the topological optimization of two-dimensional small-sized square-lattice phononic crystal simple thin-plate structures to investigate their bandgap characteristics. By combining the finite-element method with an improved genetic algorithm, the structure is optimized to achieve a maximum relative bandwidth. The transmission losses of the optimized unit-cell structures in different frequency ranges are calculated, and the algorithmic optimization design approaches for achieving a maximum relative bandwidth in high-frequency, mid–low-frequency, and ultra-low-frequency ranges are analyzed in detail.

By comparing the topology optimization structure of small-size binary phononic crystal simple thin-plates with traditional plate shapes, columns, and multi-component or large-size complex phononic crystal plate topology optimization structures, it is shown that small-size binary simple thin-plate structures can achieve the maximum relative bandwidth within different specified frequency ranges, and the low-frequency sound insulation frequency band can be as low as 34 Hz. Modal analysis of the structural vibration modes reveals that the low-frequency bandgap characteristics of the small-sized phononic crystal thin-plate structures conform to the localized resonance mechanism. Furthermore, the parameters of the constituent materials and the filling positions of the scatterers in the thin-plate structure have varying degrees of influence on the starting and ending positions and sizes of the bandgaps.

The research results of this work enrich the understanding of how to utilize algorithmic optimization design for phononic crystal structures within the desired frequency range. Experimental validations will be carried out in the subsequent work. We are in the process of building up our laboratory and will try to perform as many experiments as possible to further support our research in subsequent work. Future work provides an algorithmic design approach for the topological optimization of low-frequency bandgap characteristics in thin plate structures. Moreover, the topologically optimized two-component thin-plate structures have the advantages of simplicity and ease of processing. They can be applied in areas such as highway noise barriers or railway noise control, offering new possibilities for low-frequency and even ultra-low-frequency vibration damping and noise reduction in engineering and mechanical applications.

## Figures and Tables

**Table 1:** Parameters of filling materials for two-dimensional phononic crystal plate structures.

Material	Young Modulus *E*/10^10^ Pa	Density *ρ*/kg·m^−3^	Poisson Ratio *μ*
Tungsten	35.41	19,100	0.35
Lead	4.08	11,600	0.37
Aluminum	7.76	2730	0.35
Epoxy Resin	0.435	1180	0.37
Rubber	9.942 × 10^−5^	1600	0.47
Silicone Rubber	1.175 × 10^−5^	1300	0.47

## Data Availability

Raw data are available from the authors upon request.
